# A facile synthesis approach of silica aero-gel/eicosane particles and its potential application on polyester fabric to impart thermoregulation properties

**DOI:** 10.1016/j.heliyon.2023.e12935

**Published:** 2023-01-11

**Authors:** Moni Sankar Mondal, Syed Zubair Hussain, Mohammad Ullah

**Affiliations:** Department of Textile Engineering, Khulna University of Engineering & Technology, Khulna, Bangladesh

**Keywords:** Silica aero-gel, Phase change material, Polyester knitted fabric, Textile dyeing &coating, Thermo-regulated fabric

## Abstract

This article aims to study the thermo-regulating properties of infiltrated Phase change material (PCM) micro-particles treated on polyester fabric. The melt infiltration method was implemented for the synthesis of the Silica aero-gel/Eicosane particles by dispersing eicosane in silica aero-gel. Synthesized particles were incorporated into the polyester knitted fabric by both exhaustion dyeing and coating method to impart the thermoregulation characteristics. The crystalline structure and the particle size of aero-gel infiltrated PCM particles were measured by X-ray diffraction (XRD) analyzer. The presence of eicosane particles deposited on the fabric surface was confirmed by the Fourier Transform Infrared Spectroscopy (FT-IR) and Scanning Electron Microscope (SEM). Finally, while the sample was subjected to heating, both the dyed and coated fabric showed resistance against the rise of temperature due to the presence of phase transition PCM micro-particles compared to the untreated raw fabric sample.

## 1 Introduction

Silica aero-gel is a lightweight synthetic material with ultrahigh-high porosity and high surface area with the best thermal insulation property [1,2]. The advent of this material was primarily intended for engineering and medical applications [3,4]. In recent years, the application of aero-gel in the textile sector has increased due to its excellent thermophysiological comfort [[5], [6], [7]]. However, some intrinsic drawbacks i.e. poor durability, volumetric shrinkage, and dust release makes difficulties in the application of textile materials which need to be overcome in order to broaden the applicability of such auspicious materials [8]. Generally, the application process of Silica aero-gel on textile is done by either exhaustion dyeing process or coating process [9].

A Phase change material (PCMs) stores heat from the environment and releases heat when it is needed and turns itself into a solid [10,11]. When PCMs undergo a phase shift, it stores thermal energy, allowing temperature stabilization [12]. The direct and conventional application of the heat storage capability of PCM is found in cold weather outdoor gear and textile clothing where PCM absorbs external incoming heat flux and stores the human body heat to keep the body warm and releases it when it is required [13,14]. PCMs possess higher latent fusion heat, nearly 198J/g, and the melting point ranges from 36 °C to 38 °C, which is close to the comfortable temperature for the human body [15,16]. As a result, PCMs can be used as thermal insulators by creating an insulation barrier between the heat source and the body. Although the PCMs are not a modern phenomenon, with the renovation of modern technology, it is now possible to integrate these into different materials [17]. PCMs are incorporated into the textile substances either in the pure form or in the encapsulated form at the different stages of textiles fabrication from fiber to garment [18,19].

However, it is important to maintain the stability of PCMs in the liquid phase during its application on the textile surface [20]. The movement of PCMs needs to be prevented otherwise, it will tend to discharge from the applied surface during phase transition [21]. The stability can be controlled by the encapsulation and trapping of PCMs inside a porous media [22,23]. In the encapsulation method, stability is maintained by holding the liquid PCMs by a shell so that the infiltrated eicosane (PCM) absorbs the outward incoming heat to a certain degree to defend the body and transform its possession from solid to liquid but does not drip out at even high temperatures and becomes solid again while releasing the stored energy [24]. PCMs have been used for a long time in textiles for thermal comfort. US National Aeronautics and Space Administration used PCMs in the spacesuit in 1980 to resist drastic temperature change [25]. In another study, poly(ethylene glycol) was incorporated with the microstructure of fabric which showed slow cooling and heating rate [23]. Also, both organic (paraffin wax) and inorganic (Na_2_SO_4_.10H_2_O) PCMs were used as a coating on textiles [26,27]. The use of eicosane/aero-gel in textile material is comparatively a new concept, yet it needs to be explored to its full potential [28,29]. Eicosane is one of the widely used PCM materials widely used in textile products, changes its phase around body temperature and maintains the thermo-regulating properties for the human body's comfort [30,31]. There are various methods are available in which either microencapsulated PCM, infiltrated PCM, or pure PCM has been applied at different production levels of the textile substance, from fiber stage to completed garments [31,32].

This study describes the application of the eicosane/aero-gel micro-particles to the polyester fabric for thermoregulatory purposes in the coating and high-temperature exhaust dyeing process. Polyester is a synthetic fiber and processes a greater lifetime than other natural and synthetic fibers [33]. In the dyeing method, the polyester fabric is treated at 120 °C for 30–40 min [34]. However, the garments produced using 100% polyester fiber show lower thermal insulation properties which causes uncomfortably while wearing in the summer [35]. Air is trapped by the countless tiny polyester fibers, convection is inhibited and the air becomes a great thermal insulator [36]. As polyester traps thermal energy, the wearer gets uncomfortable. However, garments from polyester fabric are widely used blending with other natural fibers such as cotton, wool, silk, and viscose due to their cost-effectiveness [37]. The application of the infiltrated microparticle in the polyester fabric is feasible as it acts as a barrier between the sunlight and the fabric, which resist the fabric to be heated up further [38,39].

This work intends to highlight the achievement of scientific research on Silica aero-gel/Eicosane particles by melt infiltration method on polyester knitted fabric by both exhaustion dyeing and coating process and analyze the stability of infiltrated PCM at high temperatures and examines the performance against the washing and rubbing process. The size of the particle was evaluated by X-Ray Diffraction (XRD) analysis. Also, the presence of the particles was confirmed by FTIR and SEM image analysis.

## 2 Experimental methods

### 2.1 Materials

N-Eicosane as a Phase change material was purchased from Sigma Aldrich (subsidiaries of Merck Group, Germany). Silica aero-gel was collected from Brother's Garments Wholesale and Retail (New Delhi, India). In this work, 100% polyester knitted fabric (140 GSM, Tubular form) was received from Apex Holdings Limited (Savar, Bangladesh). Acrylic Binder (Printofix Binder 77 N) and Melamine based fixing agent (Printofix FixingWB) were supplied by Archroma (Bangladesh). Disperse Dye (TERASIL® BLUE W), Leveling Agent, and Dispersing Agent were purchased from Huntsman (Bangladesh). Multi-fiber fabric, rubbing cloth, Sodium perborate (NaBO_3_·_n_H_2_O), Standard detergent, Acetic acid (100%), Distilled Water, and Filter paper was obtained from the City Scientific Store, Khulna, Bangladesh. All the chemicals were labratory standard. All the chemicals were used without any further purification process.

### 2.2 Method

#### 2.2.1 Preparation of silica gel

**Fig. 1:**
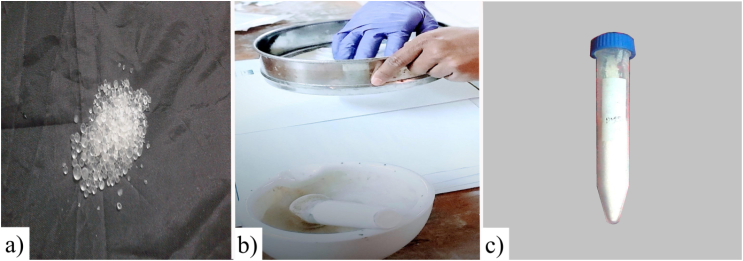
a) Sample of collected Silica gel b) Reduction of size by mortar pestle and filtration by Micro-size c) Sample of powdered size Silica gel.

At first, the collected silica aero-gel (200–250 μm) (Fig. 1a) was grained and filtrated (Fig. 1b) to obtain the desired smaller size particles for better penetration into the fabric (Fig. 1c). For this purpose, different micro-sieves that were available in the laboratory were used. After several times of processing of filtering, large-sized particles were removed and final sizes were found in fine powder form.

#### 2.2.2 Preparation of eicosane/silica-aero-gel microparticle

**Fig. 2:**
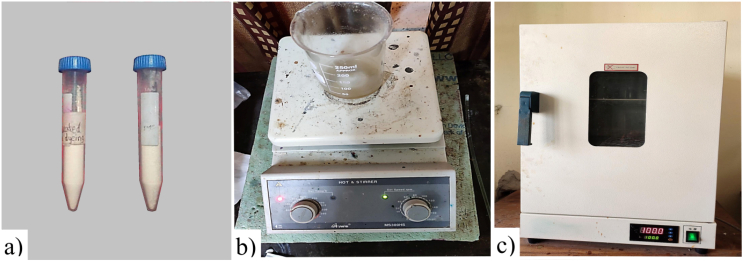
a) Sample of Micro silica aero-gel and n-eicosane b) Infiltration of n-eicosane into silica gel by Magnetic Stirrer c) Evaporation of the extra n-eicosane by Vacuum oven.

To get sufficient fluidity of liquid PCM and bring adequate infiltration into fragile aero-gel material (Fig. 2a), eicosane was heated at 80 °C which was actually double to its melting point. Simultaneously, the aero-gel particles were heated at 50 °C and mixed slowly with the molten material PCM at the ratio of 1:5.4 [40]. The mixture was continuously stirred at 80 °C with a high-speed mixer for 2 h (Fig. 2b) to avoid the accumulation of aero-gel crystals. The filter paper was used afterwards to extract the residual eicosane after 2 h, using a suction filtering system with continuous heating. Finally, a vacuum oven (Fig. 2c) was used for the long-term evaporation of excess eicosane from the surface of the aero-gel particle and obtained the required infiltrated micro-particles.

#### 2.2.3 Application of eicosane/silica-gel micro-particles in exhaust dyeing method

**Fig. 3:**
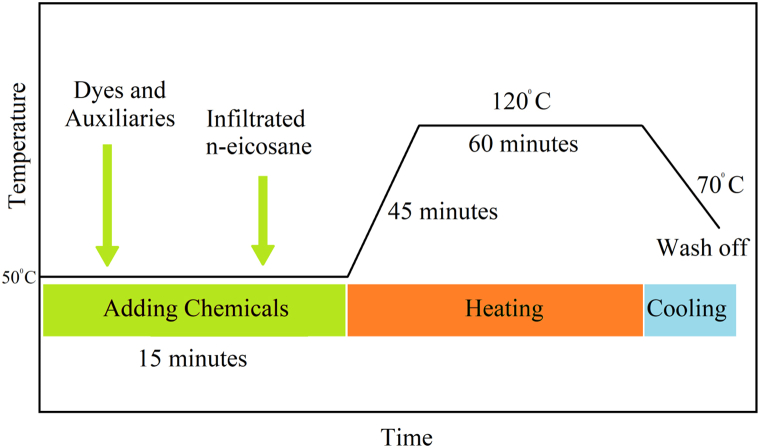
Process curve of polyester dyeing in the exhaust method.

At first, the sample fabric was weighted, and the required chemicals were calculated according to the fabric weight. Keeping the material liquor ratio 1:10, eicosane/aero-gel solid micro-particles, including all the chemicals, were taken in the canister, and the process was started at 50 °C and run for 15 min. After that, the temperature gradually increased up to 120 °C and ran for 60 min (Fig. 3) [41]. Finally, the fabric was washed at 70 °C and dried with a vacuum dryer. After dyeing and washing, fabric GSM was increased to 143.

#### 2.2.4 Application of eicosane/silica-gel microparticle by the coating method

Another approach of this work was to apply the micro-particles on the polyester knitted fabric by the dip-coating method. In this method, the fabric was immersed in the solution containing the microparticles, dyes, acrylic binder, and fixing agent (ERGOFIX-ALS). The fabric was kept for 10 min after that sample was dried at 50 °C for 5 min and cured at 140 °C for 2 min. Fabric GSM was found 146 after coating and washing.

### 2.3 Characterization and measurement

#### 2.3.1 X-ray diffraction (XRD) analysis of silica aero-gel/eicosane micro-particles

RIGACO (smart lab, Japan) was used to determine the average size of infiltrated micro-particles by applying the Scherrer equation, and 2θ was calculated using the Triclinic crystal system with CuKal radiation at wavelength 1.54059 Å. The average particle size of microparticles was measured by using the Scherrer equation. The equation is given below:

(1) τ=kλ/βcosθ

Where,

τ = mean size of the ordered (crystalline) domains
κ = dimensionless shape factor
λ = X-ray wavelength
β = line broadening at half the maximum intensity (FWHM)
θ = Bragg angle.

#### 2.3.2 Fourier transform infrared spectroscopy (FTIR)

FTIR (PerkinElmer, America) was used to characterize micro-particles on the treated fabric. For this purpose, samples were mixed with 2-propanol and pressed into a pellet, and the absorbance of the functional groups was measured within 500–4000 cm^−1^ size.

#### 2.3.3 Scanning electron microscope(SEM)

The surface morphology of the treated polyester fabric was investigated using the SEM (FEI INSPECT S50, USA) at a 500–2000 magnification range with the label of 100–400 μm of 15 KV. This test was done in Atomic Energy Center, Dhaka, Bangladesh.

#### 2.3.4 Measurement of thermal properties

The DT-8819H Series IR Laser Thermometer (temperature range −50 °C–750 °C) and Controllable guarded hotplate (ceramic coated top plate, made in Korea) were used to measure the thermo-regulating behavior of untreated fabric, and micro-particles treated dyed and coated polyester fabric samples. The air temperature was set at 24 °C to assess the sample's thermal tolerance, and the 65% relative humidity was maintained. Initially, the hot plate (temperature range 0–380 °C) was heated to a constant temperature equal to the human skin temperature, i.e. (theoretically 35 °C). Then the hot plate temperature was set at 42 °C before entering a steady state. The textile specimen was put on the center of the top plate (180 mm × 180 mm), as well as the Infrared Thermometer measured the flux of heat from the specimen into the atmosphere.

#### 2.3.5 Measurement of durability of micro-particles to washing and rubbing

Our approach was to find out the stability of micro-particles in the fabric against the wash and rubbing. For that reason, the washing process was carried out in a Gyro-wash (Gyro-wash 815, James, H Heal& Co Ltd, Halifax, England) using standard testing procedure ISO (International Organization for Standardization) 105-C06-C2S. For this test, 10 cm × 4 cm polyester was taken and carried out at 40 °C for 30 min.

The rubbing process was carried out by using the standard testing procedure ISO 105-X12 in SDL Atlas Crock-meter**-** M238A (SDL Atlas, England) at 25 °C and 65% RH. A 14 cm × 5 cm polyester fabric sample was used for the rubbing test. The temperature and the.

## 3 Results & discussion

### 3.1 XRD analysis

**Fig. 4:**
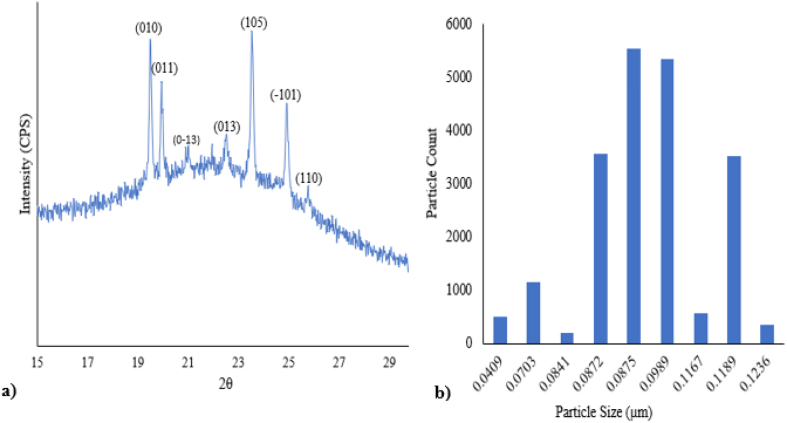
a) XRD spectra of infiltrated Eicosane/Aero-gel b) Infiltrated particle size measurement.

The XRD pattern of Silica aero-gel infiltrated PCM micro-particles is presented in Fig. 4. The diffraction peaks of the infiltrated PCM micro-particles can be represented as a well-defined triclinic crystal system with lattice constants (a = 4.32 Å and c = 4.80 Å). This pattern is observed with the gradual sharpness of the peaks where the scan range is 2Ѳ = 5–80° and step width 0.02°. The expansion of the crystalline scale is more pronounced at wide angles 2Ɵ. The entire crystallite structure is illustrated in Fig. 4a. PCM particles provide sharp peaks at 2Ɵ = 19.4°, 19.5°, 23.5°, 24.9°, 34.9°, and 39.8° due to their frequent crystallization and near molecular packaging.

The average particle size of microparticles was measured using the Scherrer equation presented in Fig. 4b within the range of 0.0409–0.1236 μm. As shown in Fig. 4b, most particle sizes are above 0.0872 μm. As the particles are in the micro-level, this may facilitate uniform dispersion into the textiles while subjected to the exhaustion dyeing and coating process.

### 3.2 FTIR spectroscopy

**Fig. 5:**
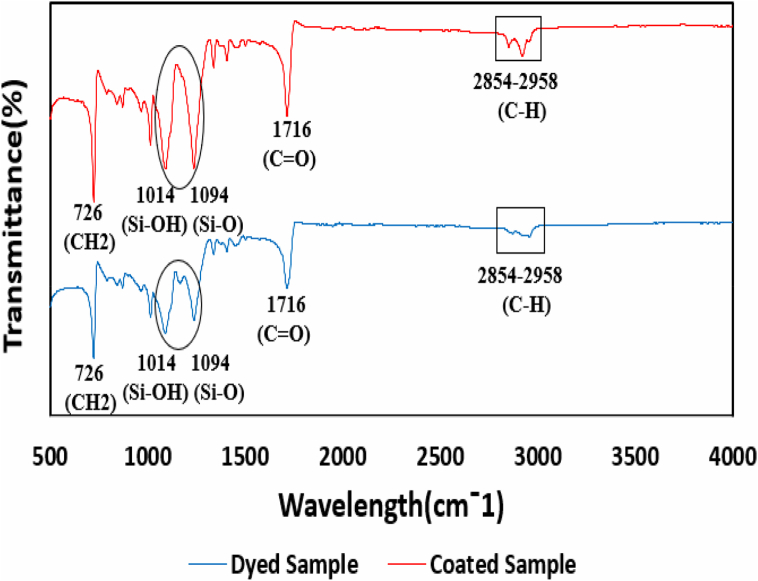
FTIR Spectroscopy of Dyed and coated fabric.

The existence of silica aero-gel/eicosane micro-particles in the treated fabric was identified with the help of the FT-IR spectrum, which is illustrated in Fig. 5. The aero-gel and eicosane peaks are marked by a circle and a box, respectively.

FT-IR spectra of micro-particles treated polyester fabric by dyeing and coating shows the peak (Fig. 5) of stretching vibration of the C–H bond in eicosane at 2854-2958 cm^−1^. The aero-gel spectra of FT-IR indicate a peak at 1014 cm^−1^ for stretching vibration of Si–OH bonds, where bending vibration of Si–O is responsible for the pick at 1094 cm^−1^. An additional –C=O stretching bond is observed at the extreme peak at 1716 cm^−1^. The 726 cm^−1^ peak seems to be the medium long-chain bond sensation of the –CH_2_ groups, which is normal in most alkanes. As shown in Fig. 5, both the FTIR spectra of dyed and coated samples exhibited almost similar peak positions.

### 3.3 Scanning electron microscopy

**Fig. 6:**
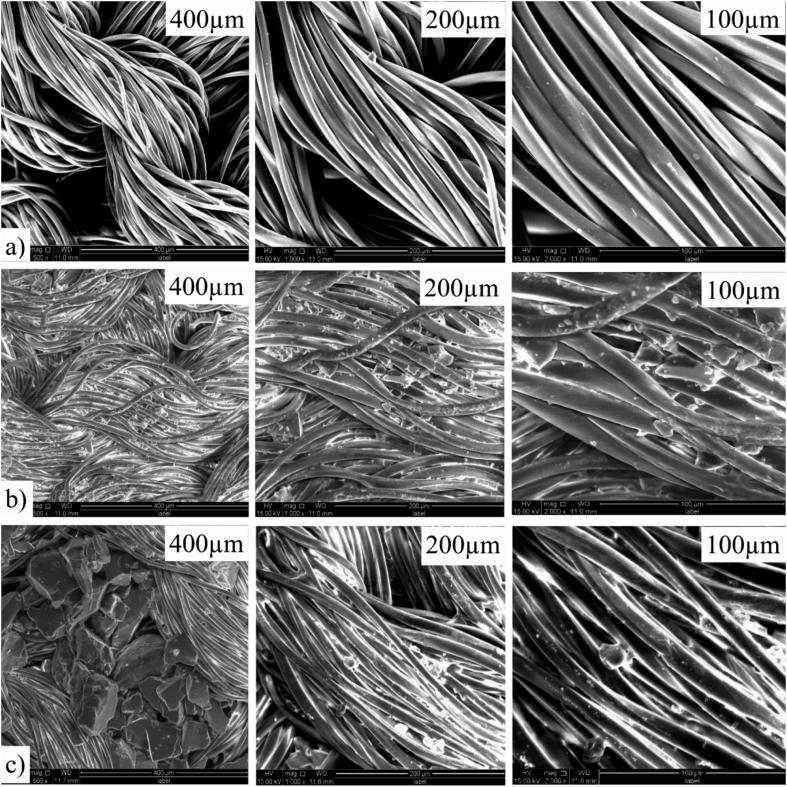
Morphological image of polyester fabric: (a) Untreated (b) Treated with PCM micro-particles by dyeing (c) Treated with PCM micro-particles by coating.

The SEM image in Fig. 6 demonstrates the presence of infiltrated PCM micro-particles on the polyester fabric. Fig. 6a shows the morphological image of untreated polyester knitted fabric. In contrast, Fig. 6b and c present PCM-treated polyester surface and indicates the slew amount of micro-particles deposited on coated fabric rather than dyed polyester fabric.

This characterisation confirms that, the coated polyester fabric would able absorb more latent heat maintaining the more thermo-regulating properties and would comply with the result of thermal analysis test of the treated fabrics.

### 3.4 Thermal analysis

#### 3.4.1 Thermal behaviour of the basic treated sample

**Fig. 7:**
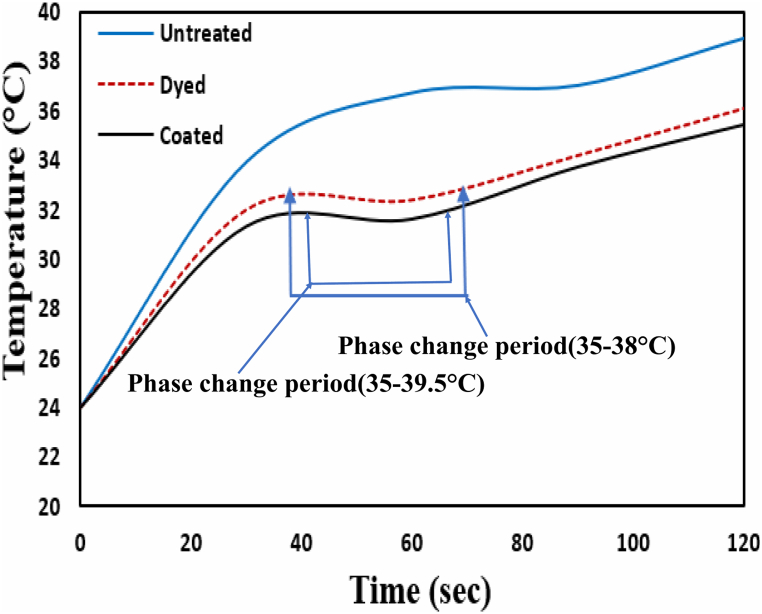
Thermo regulating properties of untreated, dyed, and coated polyester fabric.

This scientific experiment aims to analyze the impact of infiltrated PCM micro-particles on the thermoregulated behaviours of dyed and coated polyester fabric to untreated fabric presented in Fig. 7. Thermal analysis was carried out with a constant temperature of 25 °C and the Relative Humidity 65%. In Fig. 7, three samples showed an equal temperature at the beginning of the experiment, which is 24 °C. After 30 s of heat treatment, the fabric's surface temperature increased to 33.9 °C, 32 °C, and 31.3 °C for untreated, dyed, and coated samples, respectively (Fig. 7). A phase transition of the infiltrated PCM particles may commence after those temperatures, which impacts the temperature difference from untreated to dyed and coated samples. The overall phase change period of the dyed and coated samples are about 35–38 °C and 35–39.5 °C (Fig. 7), which indicates that the micro-particles are in the comfort zone with additional time allowance [40]. Hence, this result is important for regulating the thermal properties of treated fabric because the body temperature of a human is typically around 37 °C which may be different for each person. However, in every situation, the normal body temperature of a human is still within the region of 36.1–37.2 °C which will be protected by the produced micro-particles. From Fig. 7, it is observed that the temperature difference of the dyed and coated samples from untreated samples reached a maximum value of 4.3 °C and 5.1 °C, respectively, and these differences gradually became minimal until the transitional process closed to completion. So, it can be explained that the coated sample contains a higher amount of infiltrated PCM micro-particles than the dyed sample. After the phase change period, dyed and coated temperature curves maintain the gap with the untreated sample (Fig. 7). Thus, it is discernible about the thermal insulation properties of aero-gel particles which provided heat insulation and kept the temperatures of treated samples lower than the untreated sample.

#### 3.4.2 Thermal behavior of treated sample after rubbing action

**Fig. 8:**
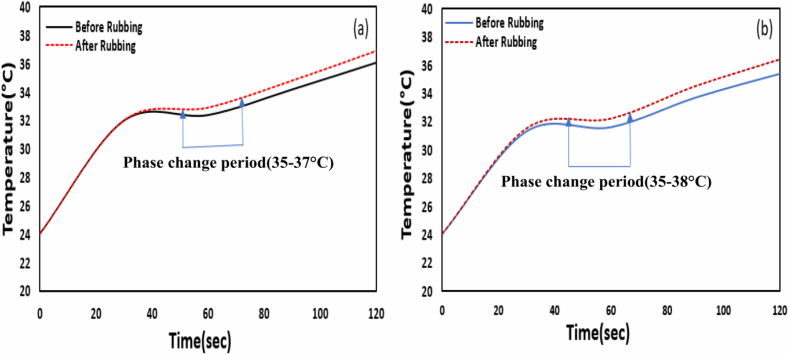
a) Thermal behavior of dyed sample before and after rubbing b) Thermal behavior of coated sample before and after rubbing.

Analysis of the stability of aero-gel infiltrated PCM micro-particles on the fabric after the rubbing process is provided in Fig. 8a and b. The dyed samples were rubbed by using the standard ISO method. The thermal testing procedure was the same as the previous method: the hotplate and the fabric temperature were set at 42 °C and 24 °C, respectively. From Fig. 8a, it can be seen that both fabric temperatures remain the same for up to 40 s and after that temperature of the rubbed sample become higher than the unrubbed sample. For example, at 60 s, the pre-rubbed sample had a temperature of 32.4 °C, whereas the post-rubbed sample had 32.9 °C. So, the difference after 60 s is 0.5 °C. Later the temperature difference increases with time (Fig. 8a). Moreover, the overall phase change period of the rubbed sample (35–37 °C) (Fig. 8a) was less than the pre-rubbed sample(35–38 °C) (Fig. 7. Thus, it can be inferred that this decrease of thermoregulation capability of the rubbed sample may be due to back out some infiltrated PCM micro-particles from the polyester knitted fabric. Fig. 8b shows the thermal curve of the pre-rubbed and post-rubbed samples of coated polyester fabric. The coated samples were rubbed as dyed fabric, and the temperature difference between rubbed and unrubbed samples followed the same trend as dyed rubbing samples. The temperature difference for the 30s, 60s, 90s, and 120s were 0.2 °C, 0.6 °C, 0.8 °C, and 1 °C respectively. Also, the coated sample's phase change duration (shown in Figs. b) decreased after rubbing from 35 to 39.5 °C to 35–38 °C. So, from this result, it can be explained that the rubbing process may damage the crosslinking bond between binder and fiber and pick out some infiltrated PCM micro-particles.

As shown in Fig. 8a and b, the shift of phase change period due to rubbing is less for the dyed sample than the coated sample where PCM was applied with a binder on the fabric. So, it can be said that the thermal resistance power of the coated sample after rubbing degrade more than dyed samples because the crosslinking bond of the coating may be hampered due to rubbing, and infiltrated micro-particles may discharge from the coated surface.

#### 3.4.3 Thermal behavior of treated sample after washing action

**Fig. 9:**
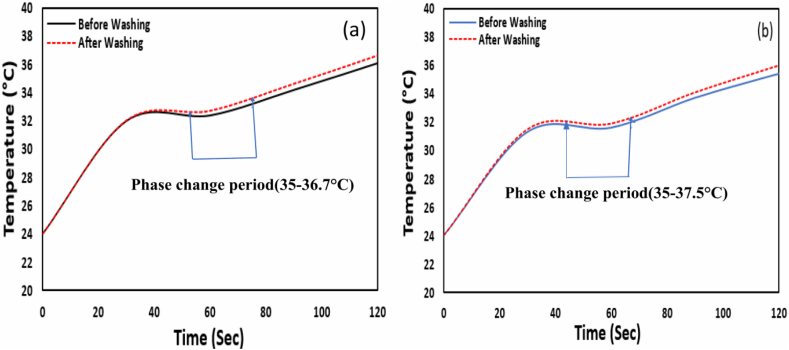
a) Thermal behavior of dyed sample before and after washing b) Thermal behavior of coated sample before and after washing.

The thermoregulation capability of infiltrated PCM micro-particles of the treated fabric after the washing process was investigated, and the results are presented in Fig. 9a and b. The washing of the dyed and coated samples was done by using the standard method. In Fig. 9a and b, the thermal curve of both dyed and coated samples remains equal to up to 40 s to the unwashed sample. After that, the temperatures of the washed sample become higher, i.e., heat resistance and thermal capability decrease more than the non-washed sample. The temperature difference between washed and unwashed samples showed the same trend as the rubbed sample, where the phase change period (Fig. 9a and b) of the dyed and coated sample was 35–36.7 °C and 35-37.5 °C**,** respectively.

The change of the phase-change period after washing was less for the dyed sample than the coated sample (Fig. 9a and b). So, it can be said that the after-washing dyed sample shows better thermal regulation capability because PCM micro-particles may discharge from the inner portion of polyester fabric at process temperature. On the other hand, aero-gel infiltrated eicosane micro-particles may discharge comparatively more from the coated surface due to damage to the crosslinking bond of the binder.

## 4 Conclusions

Eicosane/aero-gel micro-particles have been successfully applied on the polyester fabric by high-temperature exhaust dyeing and coating process where micro-particles were formed by dispersing molten eicosane in silica aero-gel. The synthesized melt infiltrated PCM micro-particles applied on the polyester fabric to analyze the thermal stability of eicosane at curing (140 °C) and investigate its applicability at high temperature (120 °C) exhaust dyeing process. As an application example, the dyed sample showed almost similar thermo-regulating properties as like coated sample, whereas the coated sample exhibited a little higher attribute than the dyeing sample. The phase change of eicosane prompted a slower increase in the temperature at about 35–38 °C and 35–39.5 °C for dyed and coated fabric, respectively, due to the variation PCM micro-particles deposited on the fabric surface, which agrees with the finding in SEM analysis. Besides, the micro-particles treated dyed samples were tested where the dyed samples showed higher thermoregulation capability after washing and rubbing compared to coated samples. The established micro-particles could be further used by varying the application method to function in various fabrics and checking the stability of thermo-regulating properties under several times washing and rubbing.

## Author contribution statement

Moni Sankar Mondal: Conceived and designed the experiments; Performed the experiments; Analyzed and interpreted the data; Wrote the paper.

Syed Zubair Hussain: Performed the experiments; Contributed reagents, materials, analysis tools or data; Wrote the paper.

Mohammad Ullah: Performed the experiments; Analyzed and interpreted the data.

## Funding statement

This research did not receive any specific grant from funding agencies in the public, commercial, or not-for-profit sectors.

## Data availability statement

Data included in article/supp. material/referenced in article.

## Declaration of interest’s statement

The authors declare no competing interests.
